# Supplementary material for: Protection and Alleviated Inflammation Induced by Virus-like Particle Vaccines Containing Plasmodium berghei MSP-8, MSP-9 and RAP1
Source: Vaccines (Basel). 2022 Jan 27;10(2):203. doi: 10.3390/vaccines10020203 (PMC8875819; doi:10.3390/vaccines10020203)
Supplement: Supplementary file 1 [file vaccines-10-00203-s001.zip › vaccines-1522827-supplementary.pdf]

Supplementary Information

Protection and alleviated inflammation induced by virus-like particle vaccines containing *Plasmodium berghei* MSP-8, MSP-9 and RAP1

Su-Hwa Lee [1], Ki-Back Chu [1,2], Hae-Ji Kang [3], Fu-Shi Quan [1,2,*]

[1] Department of Medical Zoology, School of Medicine, Kyung Hee University, Seoul 02447, Korea

[2] Medical Research Center for Bioreaction to Reactive Oxygen Species and Biomedical Science Institute, School of Medicine, Graduate School, Kyung Hee University, Seoul 02447, Korea

[3] Department of Biomedical Science, Graduate School, Kyung Hee University, Seoul 02447, Korea

* Correspondence to: fsquan@khu.ac.kr (FSQ)

Supplementary Tables

Supplementary Table S1. *Plasmodium berghei* rhoptry-associated protein 1 (RAP1) sequence comparison. Nucleotide sequences of codon-optimized (RAP1) were obtained from GenScript, and compared with the original non-codon optimized RAP1 (XM_034565422.1) sequence acquired from NCBI database.

Optimized	1	ATGTTCATCAAGATCGTGTCACTGTTCATCCTGTCCAGGCTGGTGTTCCAGGACTACTCA
Original	1	ATGTTTATTAAAATTGTAAGTTTGTTCATACTTTCCCGATTGGTGTTTCAAGATTATTCT
Translation	1	M F I K I V S L F I L S R L V F Q D Y S
Optimized	61	GTCGCTTTCAACATCAGAGACTCCAACATCATCTCCAACTACAGCCACGGTTACAACAGC
Original	61	GTGGCATTTAACATACGTGACTCTAATATTATATCCAATTATTCACATGGTTACAACAGC
Translation	21	V A F N I R D S N I I S N Y S H G Y N S
Optimized	121	CCCGGCATCAACAACGACAAGCTGGGAAACCTGAACTACTTCACCGAGATCTTCCCAAAG
Original	121	CCAGGTCTAAACAATGATAAATTAGGCAATTTGAATTATTTCACGGAAATCTTCCCCAAA
Translation	41	P G I N N D K L G N L N Y F T E I F P K
Optimized	181	AAGGAGCACGAAATCATCAACCTGTACGACGACGTGCAGGAGCTGCTGGGCAACGAACGC
Original	181	AAGGAACATGAAATTATTAATTTATATGATGATGTACAAGAATTGTTAGGAAATGAGCGA
Translation	61	K E H E I I N L Y D D V Q E L L G N E R
Optimized	241	ATGTCTTTCCTGCAGGAAGACGACGACGTCAACCAGAACAAGAACGGTACTAGCAACAAG
Original	241	ATGTCTTTTTTACAAGAAGATGATGATGTCAATCAAAATAAAAATGGAACTTCTAATAAA
Translation	81	M S F L Q E D D D V N Q N K N G T S N K
Optimized	301	TACGCTCTGTTCAACGGTAACCTGATCGCCGACCTGAAGGAGGAAGAGCCTATCGACGAA
Original	301	TACGCTTTATTTAATGGAAATTTAATAGCTGATTTAAAAGAAGAGGAACCAATTGATGAA
Translation	101	Y A L F N G N L I A D L K E E E P I D E
Optimized	361	ACCGCTGAAGAGGAAACTATCAACGAGAACATCGACGAAAAGAACACCTCAACTATCAAG
Original	361	ACCGCCGAAGAGGAAACAATTAATGAAAATATAGATGAAAAAAATACATCAACTATAAAA
Translation	121	T A E E E T I N E N I D E K N T S T I K
Optimized	421	TACACCCCCGACTACACTCCACGCCTGAAGAAGGCTATGCACACCCTGGGCTACGACAAG
Original	421	TATACACCAGATTACACACCCCGTTTAAAAAAAGCTATGCATACATTAGGCTATGATAAA
Translation	141	Y T P D Y T P R L K K A M H T L G Y D K
Optimized	481	GAGTTCAAGCTGGCCGAACTGACCACTATCCAGTCCTGCCCTAACGACAACTTCCTGTTC
Original	481	GAATTTAAATTAGCTGAATTGACAACAATACAGTCATGTCCCAATGATAATTTTTTATTT
Translation	161	E F K L A E L T T I Q S C P N D N F L F
Optimized	541	GACATCTTCCCCCAGGCCATCCAGAAGTTCCAGGAGAACGACATGAAGTACATCCAGACC
Original	541	GATATATTTCCCCAAGCAATACAAAAATTTCAGGAAAACGATATGAAATACATACAAACT
Translation	181	D I F P Q A I Q K F Q E N D M K Y I Q T
Optimized	601	CAGGGTGACCAGTACGTGGAATGCATCAAGAAGCACAAGCTGGTCGGCTCAGACAACCAG
Original	601	CAAGGGGATCAATATGTAGAATGTATTAAAAAGCATAAATTAGTTGGATCAGATAATCAG
Translation	201	Q G D Q Y V E C I K K H K L V G S D N Q

Optimized	661	GACCTGAAGCTGAACTTCGGAAACTCCGTGAACACTTTCGGTCCTTACAAGATCCCCCAG
Original	661	GACTTGAAATTGAATTTTGGTAATTCAGTTAATACTTTTGGCCCATATAAAATACCACAA
Translation	221	D L K L N F G N S V N T F G P Y K I P Q
Optimized	721	AAGATGATCACCTTCGACCTGATCCGCCTGCCAAGCAACATCACTCCTGTCAACCTGGCT
Original	721	AAAATGATTACCTTCGATTTAATCAGATTACCTTCAAATATTACTCCTGTTAATTTAGCA
Translation	241	K M I T F D L I R L P S N I T P V N L A
Optimized	781	AACGACTACTACCTGTCCGAGAGCGAGTTCCCAAACCTGCACAAGCTGAACTACTGCCTG
Original	781	AATGATTATTATTTATCCGAAAGTGAATTTCCAAATTTGCATAAACTAAATTATTGTCTA
Translation	261	N D Y Y L S E S E F P N L H K L N Y C L
Optimized	841	CTGCACCCTGCCAAGCTGGAGAAGCTGCTGAAGCGTAAGGACATCAAGTCTTACATCAAC
Original	841	TTACACCCTGCAAAATTAGAAAAATTATTAAAAAGAAAAGATATTAAATCATATATAAAT
Translation	281	L H P A K L E K L L K R K D I K S Y I N
Optimized	901	AACACCGAATCTGGATCATACGACAACTTCTTCAAGAAGGCTATGAACGAGAGCATCGAA
Original	901	AATACAGAATCTGGCTCTTATGATAATTTTTTTAAGAAGGCTATGAATGAATCCATTGAA
Translation	301	N T E S G S Y D N F F K K A M N E S I E
Optimized	961	TGCCACGTGGAGAACACCCTGCACATGATCCTGTCTAAGCTGACTCTGTTCATGTTCTTC
Original	961	TGCCATGTTGAAAATACATTACATATGATATTATCAAAACTCACACTTTTTATGTTCTTT
Translation	321	C H V E N T L H M I L S K L T L F M F F
Optimized	1021	AACGTCAACAAGCCAGACTCCAAGAACATCCTGAAGAAGCAGCTGTACATCATCAAGTCT
Original	1021	AATGTTAACAAACCAGATTCTAAGAATATCCTTAAGAAGCAATTGTACATAATAAAATCA
Translation	341	N V N K P D S K N I L K K Q L Y I I K S
Optimized	1081	GGCCTGTCATACCGCTCCCGTAAGTACGTGGACAACGCCTACAAGAAGGTCATCAACAAC
Original	1081	GGGCTAAGTTATAGAAGTAGAAAATATGTCGATAATGCTTATAAGAAGGTTATAAACAAT
Translation	361	G L S Y R S R K Y V D N A Y K K V I N N
Optimized	1141	TTCAAGGACTACGAGAACAAGATCAAGCTGATCGACAGCAACCTGGAAAACATCACCTCT
Original	1141	TTTAAAGATTACGAAAACAAAATAAAATTGATTGATTCCAATTTGGAGAATATAACTTCG
Translation	381	F K D Y E N K I K L I D S N L E N I T S
Optimized	1201	TACTACGCTGCCCACGCTTTCGGAAACCTGTGCAACACTTACATGGAGAAGGACAACATC
Original	1201	TACTATGCCGCGCATGCTTTTGGTAATTTATGCAACACATATATGGAAAAAGATAATATA
Translation	401	Y Y A A H A F G N L C N T Y M E K D N I
Optimized	1261	TACGAAGCTAACGCCTACCTGTACGAGCACATCGCCCCTAGCATCAAGATCTTCTCCAGC
Original	1261	TATGAAGCAAATGCATATTTGTATGAGCACATTGCTCCATCCATTAAAATTTTCTCTTCT
Translation	421	Y E A N A Y L Y E H I A P S I K I F S S

Optimized	1321	TGCATCAAGCACCTGACCATCTACAACTACATCATCTCAAACCTGCTGGGCCAGGTGAAG
Original	1321	TGTATTAAACATTTGACAATTTATAATTATATTATATCAAATTTACTTGGTACCGTAAAA
Translation	441	C I K H L T I Y N Y I I S N L L G Q V K
Optimized	1381	CACCTGATGTCCTACACTCCTAGGAAGCCCATCCTGAAGGACATCCACTTCAAGGCTCTG
Original	1381	CATTTAATGAGTTATACCCCTAGAAAACCAATATTAAAAGATATACATTTTAAGGCTCTT
Translation	461	H L M S Y T P R K P I L K D I H F K A L
Optimized	1441	CTGAACAAGTTCAAGAAGCCTCAGAACGTGAACGAGCTGCCATACGACCCTACCGTCAAG
Original	1441	TTAAACAAATTTAAAAAACCCCAAAATGTAAATGAACTTCCATATGACCCAACTGTTAAA
Translation	481	L N K F K K P Q N V N E L P Y D P T V K
Optimized	1501	TCATTCGCCCTGGGCGAGCTGACTAGAGAACCCATCCACGGACTGATCCACTCCTACTTC
Original	1501	TCATTTGCACTTGGTGAATTAACGAGAGAACCTATTCATGGTTTAATTCATTCGTACTTT
Translation	501	S F A L G E L T R E P I H G L I H S Y F
Optimized	1561	GAATACAAGAAGAAGGACCTGCTGGACATCATGCAGAAGCTGAAGCTGGACATCTTCAGC
Original	1561	GAATATAAGAAAAAAGACCTATTAGATATTATGCAGAAGCTCAAATTAGATATTTTTTCA
Translation	521	E Y K K K D L L D I M Q K L K L D I F S
Optimized	1621	CTGGCTAACAAGGACCTGAAGTTCCCCTCTGCCGACCTGCCAGACTACAAGCTGTTCAAG
Original	1621	CTTGCTAATAAAGATTTAAAATTTCCAAGTGCCGATTTGCCAGATTATAAATTGTTTAAA
Translation	541	L A N K D L K F P S A D L P D Y K L F K
Optimized	1681	GACATCGTGAACAAGTACAAGAAGGAGATCAAGATCCTGTTCCAGGAGATGAACAGCGAA
Original	1681	GATATAGTCAATAAATATAAAAAAGAAATCAAAATTTTATTCCAAGAAATGAATTCTGAA
Translation	561	D I V N K Y K K E I K I L F Q E M N S E
Optimized	1741	TACGTCAAGCTGTTCAAGATGCGCATCTCTGCTTTCTACCAGAAGGACTTCTTCATCTAC
Original	1741	TATGTCAAACTTTTTAAAATGCGTATTTCAGCATTCTACCAAAAGGACTTTTTTATTTAT
Translation	581	Y V K L F K M R I S A F Y Q K D F F I Y
Optimized	1801	GACCGTGTCTTCTAA
Original	1801	GATCGTGTATTCTAA
Translation	601	D R V F

Supplementary Figures

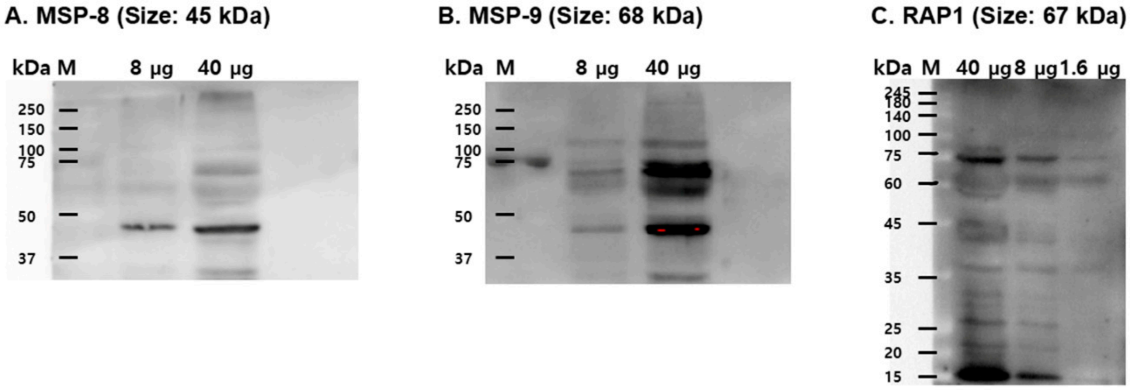

Supplementary Figure S1. The original uncropped western blot images of merozoite surface protein 8 (MSP-8, **A**), merozoite surface protein 9 (MSP-9, **B**), and rhoptry-associated protein 1 (RAP1, **C**) of *Plasmodium berghei.*
